# Total en bloc spondylectomy combined with the satellite rod technique for spinal tumors

**DOI:** 10.1186/s13018-020-02058-x

**Published:** 2020-11-16

**Authors:** Hongyu Wei, Chunke Dong, Jun Wu, Yuting Zhu, Haoning Ma

**Affiliations:** 1 Department of Orthopaedic Surgery, China-Japan Friendship Hospital, 2 Yinghuadong Road, Chaoyang District, Beijing, 100029 China; 2 Beijing University of Chinese Medicine, 11 North Third Ring East Road, Chaoyang District, Beijing, 100029 China; 3 Department of Orthopaedic Surgery, People’s Hospital of Ningxia Hui Autonomous Region, 301 Zhengyuan North Street, Jinfeng District, Yingchuan, 750002 China; 4 Beijing Tongzhou Integrative Medicine Hospital, 89 Chezhan Road, Tongzhou District, Beijing, 101100 China

**Keywords:** Total en bloc spondylectomy, Satellite rod, Spine, Primary tumor, Neoplasm metastasis

## Abstract

**Background:**

Instrumentation failure (IF) is a common complication after total en bloc spondylectomy (TES) in spinal tumors. This study aims to evaluate the clinical outcomes of TES combined with the satellite rod technique for the treatment of primary and metastatic spinal tumors.

**Methods:**

The clinical data of 15 consecutively treated patients with spinal tumors who underwent TES combined with the satellite rod technique by a single posterior approach from June 2015 to September 2018 were analyzed retrospectively. Radiographic parameters including the local kyphotic angle (LKA), anterior vertebral height (AVH), posterior vertebral height (PVH), and intervertebral titanium mesh cage height (ITMCH) were assessed preoperatively, postoperatively, and at the final follow-up. The visual analog scale (VAS), Oswestry Disability Index (ODI), and American Spinal Injury Association (ASIA) scale were used to assess quality of life and neurological function. The operative duration, volume of blood loss, and complications were also recorded.

**Results:**

The mean operation time and volume of blood loss were 361.7 min and 2816.7 mL, respectively. During an average follow-up of 31.1 months, 2 patients died of tumor recurrence and *multiple* organ metastases, while recurrence was not found in any other patients. Solid fusion was achieved in all but one patient, and no implant-related complications occurred during the follow-up. The VAS, ODI, and ASIA scores significantly improved from before to after surgery (*P* < 0.05). The LKA, AVH, and PVH significantly improved from before to immediately after surgery and to the final follow-up (*P* < 0.05), and the postoperative and final follow-up values did not significantly differ (*P* > 0.05).

**Conclusions:**

TES combined with the satellite rod technique can yield strong three-dimensional fixation and reduce the occurrence of rod breakage, thereby improving the long-term quality of life of patients with spinal tumors.

## Background

Currently, it is generally accepted that total en bloc spondylectomy (TES), in combination with multidisciplinary management, is crucial for disease-free survival in patients with primary and metastatic spinal neoplasms [1–3]. TES, which was first described by Tomita et al. [4] in 1994, has been proven to decrease the rate of local recurrence and prolong survival via a margin-free resection which can prevent tumor cell contamination in surrounding tissues [5, 6]. After en bloc resection of diseased vertebra, the spinal column is completely separated, especially in patients with tumors extending to paraspinal muscles, ribs, and other surrounding structures that also need to be removed, making the spine extremely unstable [7]. Considering that TES is indicated for patients with a longer life expectancy [8], the available longevity of spinal reconstruction is challenging for spinal surgeons to assess. It has been reported that the incidence of instrumentation failure (IF) after TES is as high as 40% [7], and the incidence of rod breakage among these cases is 37.5% [9]. Spinal instability caused by IF after TES can lead to reoperation in as many as 25% of patients due to severe pain and neurological deterioration, which is unacceptable for patients with a poor general condition and spinal tumors [9].

In recent years, the satellite rod technique, a complex of bilateral satellite rods in addition to the 2-rod construct around three-column osteotomy sites, has been widely performed for treating severe spinal deformities [10–12]. Compared with a standard 2-rod construct, the novel 4-rod technique has been confirmed to be a safe, simple, and effective method to provide increased stability and significantly prevent IF and symptomatic pseudarthrosis with the following advantages [13]: (1) the multi-rod construct shares the stress at the osteotomy site [10], (2) control the closing of the osteotomy to reduce the risk of vertebral body translation [14], (3) help to better maintain balance in the coronal and sagittal planes [14], and (4) convenient and simple to add satellite rods through a double U head connector [14].

To the best of our knowledge, few studies have developed a surgical protocol based on TES combined with the satellite rod technique for the treatment of spinal tumors. Herein, the current study employed satellite rods across the osteotomy site and aimed to demonstrate the feasibility and safety of the satellite rod technique for TES in spinal tumors.

## Materials and methods

### Patients

From June 2015 to September 2018, 15 consecutively treated patients suffering from spinal tumors who underwent TES and the satellite rod technique in our department were included. There were 7 males and 8 females with an average age of 45.5 years (range, 23~73 years). All cases were pathologically confirmed by preoperative CT-guided biopsy. Of the 15 cases, 9 involved primary tumors (4 giant cell tumors, 2 plasmacytomas, and 3 aggressive hemangiomas) and 6 involved metastatic tumors (primary organs including the lung in 2 cases, breast in 1, liver in 1, thyroid in 1, and prostate in 1). The inclusion criteria were as follows: (1) diagnoses of solitary primary spinal tumor or metastatic tumor confirmed by preoperative computed tomography (CT), magnetic resonance imaging (MRI), and positron emission tomography-computed tomography (PET-CT); (2) tumors that satisfied the criteria for Tomita’s classification types I~V [15]; and (3) preoperative results for the revised Tokuhashi scoring system [16] for the prognosis of the metastatic spinal tumor, and a survival time of the patients of more than 6 months. The patients’ clinical data and tumor characteristics are summarized in Table 1.

**Table 1:** Patients’ clinical data and tumor characteristics

Parameters	
*Age (years)*	45.5 (23~73)
*Gender (male/female)*	7/8
*Previous treatment (n)*	
Surgery for primary tumor	5
Chemotherapy	6
Radiotherapy	2
None	5
*Primary tumors (n = 9)*	
Giant cell tumors	4
Plasmacytomas	2
Aggressive hemangiomas	3
*Metastatic tumors (n = 6)*	
Lung	2
Breast	1
Liver	1
Thyroid	1
Prostate	1
*Tumor location (n = 15)*	
T1	1
T3	1
T8	1
T9	1
T11	2
T12	5
L1	4
*Tomita’s classification (n = 15)*	
II	4
IV	7
V	4
*Revised Tokuhashi score for metastasis (n = 6)*	
9~11	3
12~15	3

### Operative procedures

**Fig. 1:**
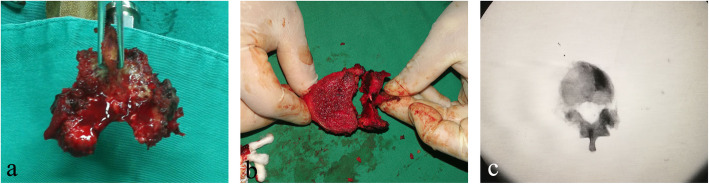
TES technique. **a** En bloc dorsal elements. **b** En bloc resection of the ventral vertebral body. **c** Radiograph showing the complete removal of the diseased vertebra

Somatosensory-evoked potentials (SEPs) and motor-evoked potentials (MEPs) in the spinal cord were monitored throughout the entire surgical procedure. After general anesthesia was induced, the patient was placed in a prone position on an adjustable spinal frame. Under C-arm X-ray guidance, the spine was exposed through a standard posterior midline approach with subperiosteal stripping. Then, pedicle screws were placed 2~3 levels above and below the diseased vertebra via the freehand technique, and bone cement-augmented screws were implanted in osteoporosis patients. In the thoracic spine, the dorsal part of the ribs adjacent to the costotransverse joint of the ribs was removed so that the ventral side of the diseased vertebrae could be reached. The TES technique consists of two steps—en bloc resection of the dorsal elements of the involved vertebra after transpedicular osteotomy and subsequent en bloc resection of the ventral vertebral body (Fig. 1):

*Step 1*. Laminectomy was performed in the adjacent segments above and below the diseased vertebra to expose the spinal canal. Simultaneously, the superior and inferior articular processes of the adjacent vertebrae were removed. Then, the bilateral pedicles were cut with a wire saw. After en bloc dorsal procedures were performed, the bone surfaces of the cut section were immediately sealed with bone wax to reduce bleeding and minimize tumor contamination.

*Step 2*. The adjacent intervertebral disk and posterior and anterior longitudinal ligaments were cut off carefully with an L-like dissector, curette, and rongeur. After ligation of bilateral segmental arteries, mild and blunt dissection was performed at the interface between the anterior part of the diseased vertebra and the pleura, aorta, and iliopsoas muscle in the thoracic and lumbar spine, respectively. In the thoracic spine, the unilateral nerve root was ligated and cut in most cases. Before the en bloc ventral vertebral body procedure was performed, a temporary stabilizing rod was fixed on one side of the pedicle screws to prevent spinal cord injury. After the spinal cord was gently peeled from the surrounding epidural venous plexus and ligamentous tissue in the spinal canal via a thin nerve dissector, the entire vertebra was rotated from the contralateral side of the unilateral internal fixation region.

**Fig. 2:**
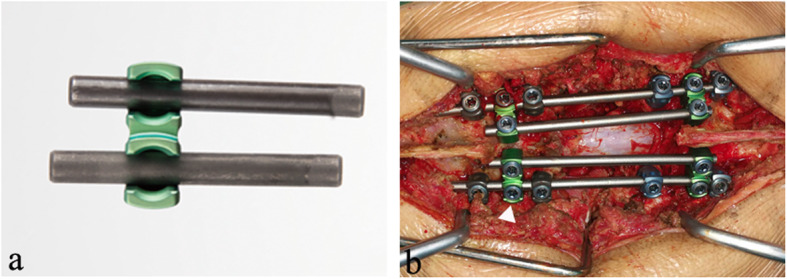
Satellite rod technique. **a** A double U head connector. **b** Satellite rods span only the osteotomy site (white triangle)

Anterior stabilization was established using a TMC (Fule Science & Technology Development Co., Ltd, Beijing, China) filled with a cancellous bone graft or bone substitutes. Posterior reconstruction of the spine was performed by a standard 2-rod instrument with affixed satellite rods (Fule Science & Technology Development Co., Ltd, Beijing, China). The two additional rods were placed medially or laterally to the original longitudinal rods via a double U head connector (Fig. 2a). Many reports [17–19] have noted the occurrence of rod failure at the level of the osteotomy and typically occurred relatively early in the postoperative period; thus, the two additional rods span only the osteotomy site (Fig. 2b). Considering that all patients included were single segment lesions, the criteria for insertion were similar at dorsal and lumbar level.

Finally, the operative field was soaked in distilled water and then 0.5 mg/mL cisplatin for 2.5 min to reduce tumor implantation.

### Postoperative management

The patients were allowed to walk 3~5 days after surgery, and rehabilitation management was recommended and performed for every patient in the first 3 months after the operation. An orthosis was used for at least 3 months until complete bone fusion was achieved. Adjuvant therapies are also performed depending on the type of pathology.

### Radiographical assessment

**Fig. 3:**
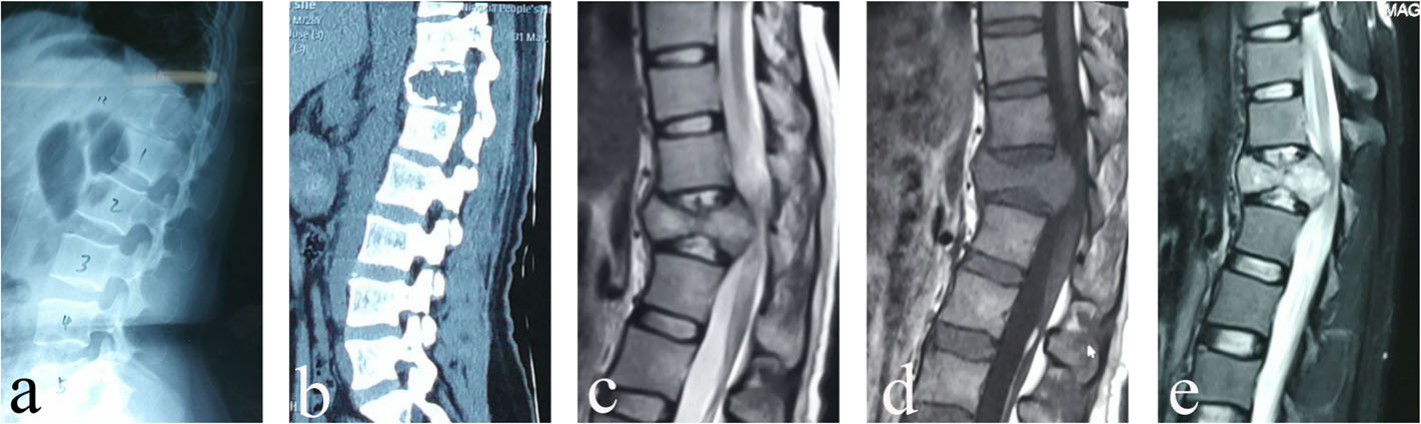
A 28-year-old female patient with a T12 giant cell tumor complicated by neurological deficits. The preoperative sagittal X-ray (**a**), CT (**b**), T2-weighted MRI (**c**), T1-weighted MRI (**d**), and STIR MRI (**e**) scans showed a pathological fracture of the T12 vertebral body with spinal cord compression

Radiography, CT, and MRI data were collected preoperatively (Fig. 3), and follow-up imaging evaluations were performed every 3 months during the first year and semiannually thereafter. The anterior vertebral height (AVH), posterior vertebral height (PVH), and local kyphotic angle (LKA) were assessed before and after surgery, as described in our previous study [20]. The intervertebral titanium mesh cage height (ITMCH) (the height of the fused segments) was measured at the midportion of the adjacent upper and lower endplates after the operation and at the last follow-up. The postoperative fusion criteria were based on the fusion classification system proposed by Brantigan et al. [21]. To correct for the magnification ratio on radiographs acquired preoperatively and postoperatively, we used a picture archiving and communication system (PACS) (Carestream Health, Inc. Shanghai, China) to assess the imaging data in our hospital; the average of the two measurements obtained by two independent senior spine surgeons was used.

### Clinical evaluation

The VAS and Oswestry Disability Index (ODI) were used to evaluate the clinical functional results before surgery and during the follow-up period. The American Spinal Injury Association (ASIA) grading system was used to assess the neurological status preoperatively and at the final follow-up. The operative duration, blood loss, complications such as intraoperative injuries to the spinal cord and dura, postoperative complications including infection, and IF were also recorded.

### Statistical analysis

All analyses were performed using SPSS 20.0 software (SPSS Inc., Chicago, USA). All data are expressed as the means ± standard deviations (SDs) for parametric analyses. Paired *t* tests were used to compare clinical data changes in the normally distributed values before and after surgery. Normally distributed data recorded at different time intervals were compared by repeated measures analysis, and non-normally distributed data were compared with the Kruskal-Wallis test. The chi-square test was used to compare count data. A value of *P* < 0.05 was considered to indicate a statistically significant difference.

## Results

### Surgical results

**Fig. 4:**
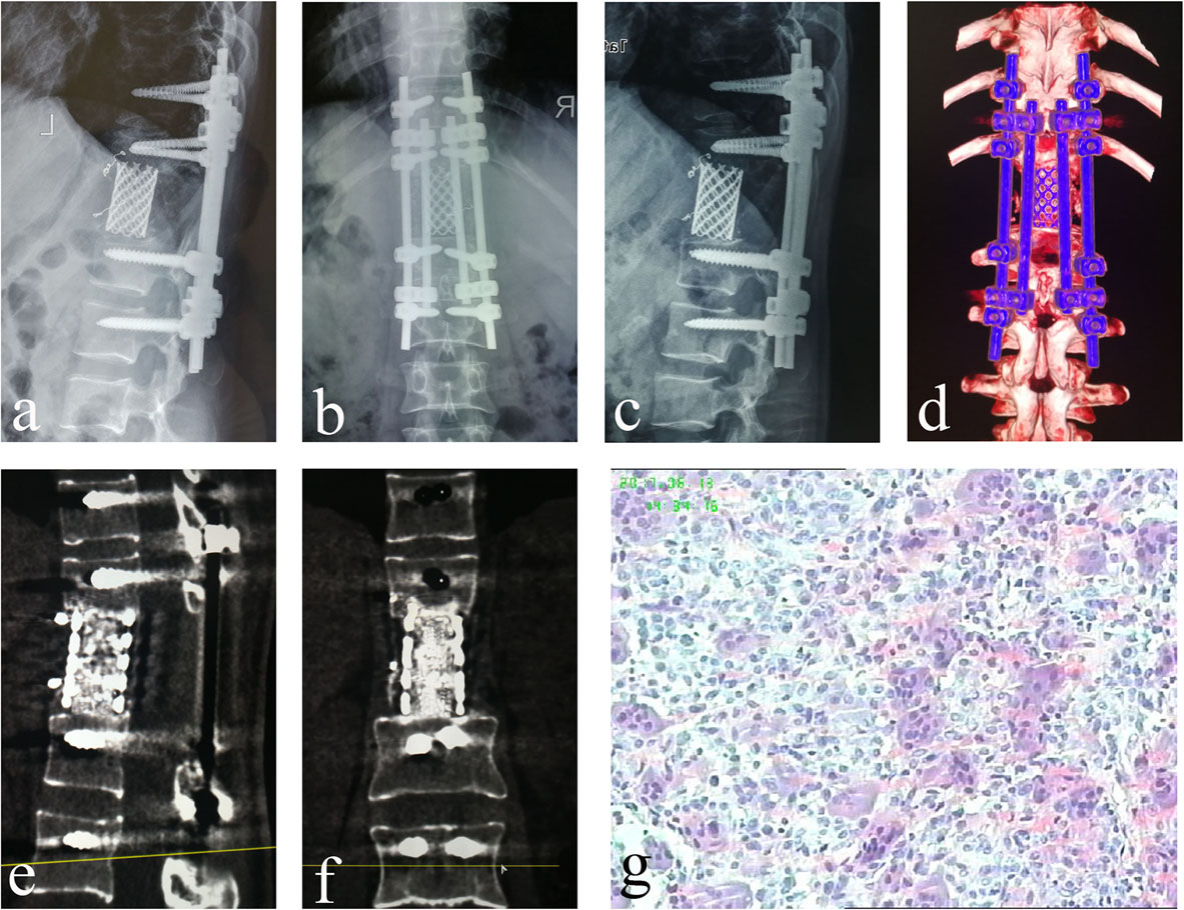
The patient underwent TES with satellite rod technique in T12. **a**, **b** The immediately postoperative plain radiographs showed corpectomy, screw fusion, an increased AVH, and restored kyphosis. **c**–**f** The plain radiographs and 3D, coronal and sagittal CT scans showed the absence of instrumentation failure, tumor recurrence, and TMC subsidence at the final follow-up. **g** The pathological results showed a giant cell tumor (H&E stain, × 20)

The average operating duration was 361.7 ± 93.76 min, with a range of 230~630 min. The mean blood loss was 2816.7 ± 1238.76 mL, with a range of 1000~5500 mL. The average number of fixed segments was 5.9. Two satellite rods were implanted in each patient. All patients underwent rehabilitation exercises by sitting up or walking 3~5 days after the operation. The average follow-up was 31.1 ± 12.33 months, with a range of 6~50 months. The postoperative adjuvant therapies included bisphosphonates, chemotherapy, radiotherapy, surgery for the primary tumor, targeted therapy, and androgen-deprivation therapy. Two patients died of tumor recurrence and *multiple* organ *metastasis*, while recurrence was not found in any of the other patients. Dural tears with cerebrospinal fluid leakage occurred in 1 patient; the tears were covered intraoperatively by fascial tissue, and a lumbar drainage tube was placed and removed after 7 days. One patient had an incision infection, 2 had a urinary tract infection, and 1 had pneumonia; all of these cases improved after symptomatic treatment with antibiotics. After the operation, 1 screw penetrated the lateral wall of the pedicle, and 3 screws penetrated the medial wall of the pedicle. There were no nerve or spinal cord symptoms, so no patients underwent related treatment. No cases of IF, such as screw loosening or rod and screw breakage, occurred at the final follow-up. The surgical results are shown in Table 2, and an illustrative case is shown in Fig. 4.

**Table 2:** Surgical results

*Operating duration (min)*	361.7 ± 93.76
*Blood loss (mL)*	2816.7 ± 1238.76
*Follow-up (months)*	31.1 ± 12.33
*Average fixed segment (n)*	5.9
*Mean satellite rod (n)*	1.7
*Complication (n)*	
Cerebrospinal fluid leak	1
Superficial wound infection	1
Urinary tract infection	2
Pneumonia	1
*Postoperative adjuvant therapy (n)*	
Bisphosphonates	8
Chemotherapy	3
Radiotherapy	1
Surgical for primary tumor	1
Targeted therapy	2
Androgen-deprivation therapy	1
*Tumor recurrence (n)*	2

### Clinical results

**Fig. 5:**
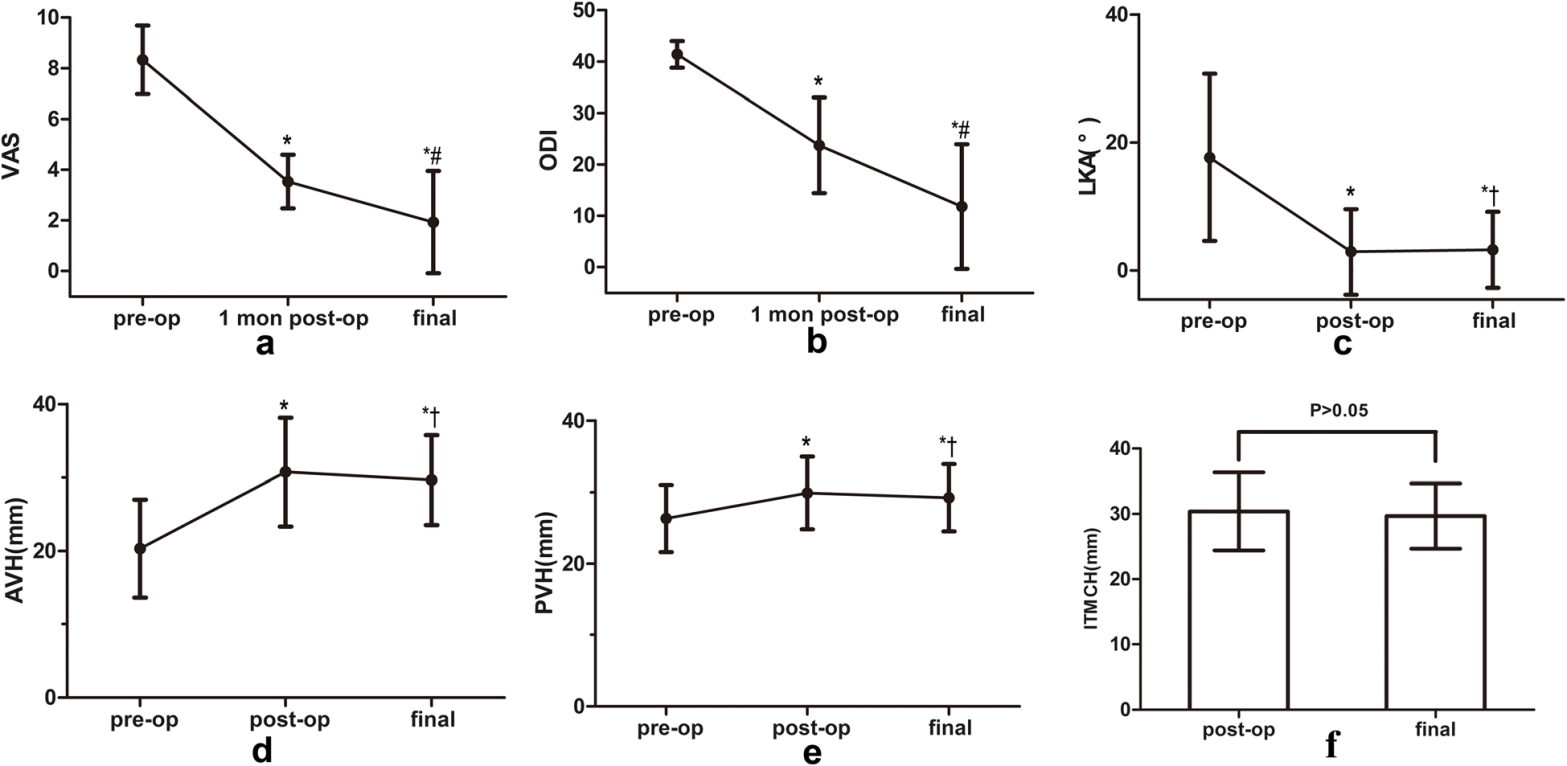
Clinical and radiological results. **a**–**e** *P < 0.05 compared with the preoperative data. **a**, **b** ^#^*P* < 0.05 compared with the 1-month postoperative data. **c**–**e** ^†^*P* > 0.05 compared with the postoperative data

All patients except 1 with neurological deficits showed improvement, and there was a significant difference in ASIA between preoperation and final follow-up (*P* < 0.05), as shown in Table 3. The VAS score decreased from 8.33 ± 1.35 preoperatively to 3.53 ± 1.06 at 1 month after operation, and to 1.93 ± 2.02 at final follow-up (*F* = 26.271, *P* < 0.001, Fig. 5a). The ODI score decreased from 41.40 ± 2.59 preoperatively to 23.73 ± 9.31 at 1 month after operation, and to 11.80 ± 12.15 at the final follow-up (*F* = 26.533, *P* < 0.001, Fig. 5b).

**Table 3:** Comparison of the preoperative and postoperative neurological statuses

	ASIA				
	A	B	C	D	E
Preoperation	0	2	8	2	3
Final follow-up	0	1	1	3	10
*χ*^2^	9.787				
*P*	0.013

### Radiological findings

Postoperative correction was immediately achieved in all the patients. The LKA changed from 17.67 ± 13.06° preoperatively to 2.92 ± 6.67° postoperatively and 3.23 ± 5.96° at the final follow-up (*F* = 34.920, *P* < 0.001, Fig. 5c). The AVH changed from 20.31 ± 6.68 mm preoperatively to 30.74 ± 7.41 mm postoperatively and 29.65 ± 6.12 mm at the final follow-up (*F* = 43.380, *P* < 0.001, Fig. 5d). The PVH changed from 26.31 ± 4.7 mm preoperatively to 29.89 ± 5.08 mm postoperatively and 29.21 ± 4.69° at the final follow-up (*F* = 11.774, *P* < 0.001, Fig. 5e). No obvious loss of correction or progressive kyphosis occurred, as there was a lack of significant differences in the parameters between the postoperative and final follow-up times (*P* > 0.05). No TMC subsidence was observed, as there was no significant difference in the ITMCH between the postoperative and final follow-up times (30.38 ± 5.97 mm vs. 29.68 ± 5.01 mm, *t* = 1.437, *P* = 0.173, Fig. 5f). Solid fusion was achieved in all patients but 1 at the final follow-up, according to the radiological evidence.

## Discussion

Because the anatomical structure of the spine is complex, radical spinal oncology resection for spinal neoplasms is nearly impossible [22, 23]. Although piecemeal or intralesional excisions may yield the complete resection of tumors, these procedures are associated with a high risk of local recurrence due to tumor contamination and residual tumors [24]. It is, therefore, advantageous to use intralesional curettage in combination with adjuvant therapies, such as radiation therapy, to achieve local control in bone metastases [25]. However, these adjuvants are not generally suitable for the spinal axis due to the risk of iatrogenic injury to neural elements and adverse effects on wound healing and bone fusion [2].

It has been confirmed that TES with wide/marginal surgical margins performed for solitary spinal metastasis and aggressive primary spinal neoplasms can yield excellent tumor control and prolong survival. Cloyd et al. [6] systematically reviewed the literature on TES, including 229 primary and 77 solitary metastatic tumors of the spine. The results showed that the 1-, 5-, and 10-year disease-free survival rates of the primary tumors were 92.6%, 63.2%, and 43.8%, respectively, and those of the metastatic tumors were 61.8%, 37.5%, and 0%, respectively. The 1-, 5-, and 10-year survival rates of the primary tumors were 96.3%, 82.2%, and 71%, respectively, and the 1- and 5-year survival rates of the metastatic tumors were 80.8% and 56.6%, respectively [6]. Compared with piecemeal or intralesional excisions, TES is currently a more suitable surgical procedure yielding good long-term local control and longer survival rates for patients with spinal tumors and favorable circumstances, according to physicians, surgeons, and patients [5, 24, 26].

Considering that TES is indicated for patients with a longer life expectancy [8], solid spinal reconstruction is essential for the long-term quality of life of patients due to its longevity [23]. A biomechanical study demonstrated that multi-segmental posterior fixation with anterior column reconstruction can provide better stability than short fixation and that fixation should be extended to 2~3 levels above and below the resected vertebrae [23]. However, instrumentation failure, the incidence of which varies from 17.0 to 40%, is not a rare complication following a long-segment fixation procedure after TES [7, 13, 27, 28]. Among the types of IF, such as screw loosening, screw back-out, cage breakage, screw fracture, and rod breakage, rod fracture is the most common and often leads to a high reoperation rate due to aggravating back pain or neurologic deterioration [9, 27]. Cage subsidence, a history of radiotherapy, and a low spinal level of involvement have been confirmed to be risk factors related to rod fracture [7, 9, 27, 28]. The survival time of spinal tumor patients is longer when primary tumors and metastatic tumors are resolved, so reconstruction of the three columns of the spine with additional posterior column reconstruction is very important [23]. The satellite rod technique, a multi-rod construct, has been proven to have the potential to reduce pseudarthrosis and fatigue fracture at the 3-column osteotomy site by enhancing the stability and stiffness of the construct for adult spinal deformity correction. Hyun et al. [10] used a multiple-rod construct, placing additional supportive rods across the 3-column osteotomy, to replace the standard 2-rod construct, resulting in a lower incidence of rod failure. Shen et al. [29] reviewed “dual construct” for 36 complex spinal reconstructions and found that the dual construct is a safe alternative to traditional 2-rod, which could avoid revision surgery after rod breakage. Some surgeons also attempted to connect additional rods to the broken rods in a reoperation for spinal tumors [14]. This practice can serve as a reminder to add satellite rods during primary TES, and in this study, we routinely used satellite rods across the osteotomy area in patients with spinal tumors undergoing TES.

In our study, reconstruction with a posterior multi-rod construct combined with anterior TMC was performed in all patients, and instrumentation failure did not occur in the postoperative follow-up. We attributed this excellent result to the following factors: (1) Posterior instrumented fusion across the apex creates long lever arms and generates substantial stress on the apical osteotomy sites. Satellite rods can disperse the stress of each rod at the osteotomy site and create a gradual transitional zone from the osteotomy area to the non-instrumented region [12]. (2) TMC subsidence prevents load sharing in the anterior column, which increases the load on the posterior fixation area and finally leads to the occurrence of a broken rod [7]. The use of additional accessory rods increases the stiffness of the instrumentation, which results in improved load transfer to the posterior construct, thereby reducing the load acting on the anterior device [13]. This factor may be the reason that TMC subsidence did not occur during the follow-up in our study. (3) Achieving solid bone fusion was vital to maintaining long-term stability for spinal reconstruction [27]. In the presence of pseudarthrosis, rod breakage may be inevitable due to metal fatigue caused by repeated fretting [27]. According to Wolff’s laws, mechanical forces influence bone formation and remodeling [30]. The stability and stiffness of satellite rods allow early rehabilitation after TES, enabling enough strain to form at the fracture site to stimulate bone formation and prevent excessive strain, which results in instability followed by delayed healing or nonunion [13]. In our cases, the overall solid fusion rate was 93.3% after a mean of 31.1 months after the surgery. Only one patient failed to obtain solid fusion because of tumor recurrence, and the patient died 6 months after the operation. (4) Radiotherapy, as a common preoperative and postoperative adjunctive therapy for spine tumors, has been proven to be a risk factor for IF after TES [27]. In this research, we obtained a wide/marginal surgical margin to minimize tumor contamination and reduce the necessity for radiotherapy after surgery. Considering the patients’ long-term quality of life and the negative impact on bone quality and healing process of radiotherapy, in our study, only 2 patients and 1 patient underwent radiotherapy preoperatively and postoperatively, respectively, after careful treatment planning.

In the retrospective study, the postoperative VAS, ODI, and ASIA scores improved significantly compared to the preoperative values (*P* < 0.05). No loss of AVH or PVH or LKA progression was observed at the final follow-up. No reoperations were performed because of IF. These results show that TES combined with the satellite rod technique can improve the long-term quality of life of spinal tumor patients. All patients were routinely fixed with 2~3 vertebral levels above and below the diseased vertebrae. The average operation time was 316.7 min, and the average volume of blood loss was 2816.7 mL. In our study, there was only one superficial wound infection, and we postulate that the infection was not caused by the satellite rod. Therefore, this technique likely does not increase the infection rate, especially considering the short time required for positioning the satellite rod via a connector. Compared with the procedures in previous studies [6, 31, 32], the procedure did not significantly increase the number of fixed segments, operation time, volume of blood loss, or complications, although 2 additional rods were implanted. From the perspective of efficiency, procedure in which satellite rods are added is relatively safe, quick, and well controlled. Furthermore, satellite rods were cut from the long rod used in the operation, so this procedure did not increase the financial burden of patients. In addition, to prevent IF, several surgical techniques such as meticulous endplate preparation [9], TMC in the oblique position [27], and disk-to-disk cutting [28] are recommended.

Despite all its strengths, there may be some shortcomings about satellite rod technique. The multi-rod construct increases the metal bulk, which could result in additional metal artifact with postoperative imaging [13]. The increased space occupation by the lateral and posterior multi-rod construct could also affect wound closure and osseous healing process [13, 33], due to proportionally less space available for placement of graft material [33]. Furthermore, the subordinate rods are piggybacked off of the primary two longitudinal rods; as a consequence, the stress transfers distally or proximally, and eventually, the primary rod is prone to break above or below the satellite rod, although this did not occur in our research [29, 33, 34].

The present study has several limitations. First, the cases included in this study were single vertebral lesions, and the effect of the satellite rod technique in multiple TES remains to be further discussed. Second, TES at the lumbar spine had the highest risk of rod fracture compared with thoracolumbar and thoracic levels. In our study, the sample size for lumbar TES, especially lower lumbar TES, was small, which can limit the statistical power. Third, because of the limited sample size, we did not establish a control group, and a prospective randomized controlled trial with a large sample size is needed to verify the reliability of the results in our study.

## Conclusions

The advantages of satellite rods include improved stability and stiffness, weight-bearing ability, and biomechanical stress dispersion, which make them extremely beneficial for preventing the occurrence of TMC subsidence, pseudarthrosis, and rod breakage. TES combined with the satellite rod technique is easy to perform, safe, and effective in improving the long-term quality of life in patients with spinal tumors.

## Data Availability

The datasets analyzed during the current study are available from the corresponding author on reasonable request.

## Abbreviations

TES: Total en bloc spondylectomy
LKA: Local kyphotic angle
AVH: Anterior vertebral height
PVH: Posterior vertebral height
ITMCH: Intervertebral titanium mesh cage height
VAS: Visual analog scale
ODI: Oswestry Disability Index
ASIA: American Spinal Injury Association
IF: Instrumentation failure
CT: Computed tomography
MRI: Magnetic resonance imaging
PET-CT: Positron emission tomography-computed tomography
TMC: Titanium mesh cage
